# Evidence of Augmented Intrarenal Angiotensinogen Associated With Glomerular Swelling in Gestational Hypertension and Preeclampsia: Clinical Implications

**DOI:** 10.1161/JAHA.119.012611

**Published:** 2019-06-25

**Authors:** Hiten D. Mistry, Lesia O. Kurlak, David S. Gardner, Ole Torffvit, Alastair Hansen, Fiona Broughton Pipkin, Helena Strevens

**Affiliations:** ^1^ Division of Child Health, Obstetrics & Gynaecology School of Medicine University of Nottingham United Kingdom; ^2^ School of Veterinary Medicine and Science University of Nottingham United Kingdom; ^3^ Primary Care Unit Lindsdal, Kalmar Sweden; ^4^ Department of Pathology Herlev University Hospital Herlev Denmark; ^5^ Department of Obstetrics Skåne University Hospital Lund University Lund Sweden

**Keywords:** angiotensinogen, glomerular, hypertension, kidney, pregnancy, urine, ACE/Angiotension Receptors/Renin Angiotensin System, Basic Science Research, Physiology, Preeclampsia, Hypertension

## Abstract

**Background:**

AGT (angiotensinogen) synthesis occurs in renal proximal tubular epithelial cells, independent from systemic AGT, as a component of the intrarenal renin–angiotensin system. We investigated urinary AGT, as a biomarker for renin–angiotensin system activation, and electrolyte concentrations, in relation to glomerular volume, as a proxy for glomerular endotheliosis in renal biopsy tissue from pregnant normotensive control and hypertensive women.

**Methods and Results:**

Urine samples were collected from normotensive control (n=10), gestational hypertensive (n=6), and pre‐eclamptic (n=16) women at the time a renal biopsy was obtained. Samples were collected from Lund University Hospital between November 1999 and June 2001. Urinary AGT, potassium, and sodium were measured, normalized to urinary creatinine. Mean glomerular volume was estimated from biopsy sections. AGT protein expression and localization were assessed in renal biopsies by immunohistochemistry. Urinary AGT concentrations were higher in hypertensive pregnancies (median, gestational hypertension: 11.3 ng/mmol [interquartile range: 2.8–13.6]; preeclampsia: 8.4 ng/mmol [interquartile range: 4.2–29.1]; normotensive control: 0.6 ng/mmol [interquartile range: 0.4–0.8]; *P*<0.0001) and showed a positive relationship with estimated mean glomerular volume. Urinary potassium strongly correlated with urinary AGT (*P*<0.0001). Although numbers were small, AGT protein was found in both glomeruli and proximal tubules in normotensive control but was present only in proximal tubules in women with hypertensive pregnancy.

**Conclusions:**

This study shows that pregnant women with gestational hypertension or preeclampsia have increased urinary AGT and potassium excretion associated with signs of glomerular swelling. Our data suggest that the kidneys of women with hypertensive pregnancies and endotheliosis have inappropriate intrarenal renin–angiotensin system activation, which may contribute toward the pathogenesis of hypertension and renal injury.

**Clinical Perspective**

**What Is New?**

- To the best of our knowledge, this study is the first to investigate, both functionally and histologically, intrarenal angiotensinogen in normal and hypertensive human pregnancies.

**What Are the Clinical Implications?**

- Monitoring urinary angiotensinogen in preeclampsia as a marker of an inappropriately activated renin–angiotensin system might be a useful indicator of renal injury and its progression both pre‐ and postpartum.

## Introduction

The renin‐angiotensin system (RAS) was originally described as a classical endocrine system, meaning that hormones are produced by glands in one organ and have remote effects elsewhere in the body after being transported by the circulatory system.^1^ However, it is now known that several tissues have autonomous or quasi‐autonomous RAS, among them the placenta and kidney.^2^, ^3^ In the proximal tubules of the kidney, all components of the RAS necessary for Ang II (angiotensin II) synthesis (ie, renin, AGT [angiotensinogen], and ACE [angiotensin‐converting enzyme]) are present, together with the downstream effectors of RAS activation, Ang II type 1 receptor (AT1R) and AT2R.^4^

During pregnancy, considerable homeorhetic responses are activated to enable the expansion of blood volume during early gestation that facilitates perfusion of the placenta and fetal growth.^5^ To allow for such dynamic changes in circulatory volume, concomitant adaptations are made in hormonal axes that regulate blood volume, including the RAS, which is activated in the first few weeks of pregnancy.^6^ The effects of continuing progesterone synthesis by the corpus luteum after a conception cycle, if unchecked, would result in sodium loss because of competitive binding to the structurally similar aldosterone receptor MR (mineralocorticoid receptor). However, early sodium loss is recognized by the macula densa and the RAS becomes activated, in turn stimulating adrenal cortical synthesis of aldosterone and improved sodium retention. Increased estrogen synthesis also stimulates hepatic AGT synthesis. In addition, during pregnancy, Ang II itself further enhances sodium retention through a direct action in the proximal tubule. There, Ang II is able to mediate through AT1R both increased uptake of circulating Ang II and augmentation of AGT expression, which can lead to local formation of Ang II and spillover of AGT excretion into the urine, where it can be measured as a marker of intrarenal RAS activity. In addition, renin secretion from principal cells of the collecting ducts can be increased by AT1R activation, and the activity of renin and/or prorenin can be enhanced by binding to the (pro)renin receptor either on intercalated cells or in soluble form. All described feed‐forward mechanisms can, together, augment intratubular and renal interstitial Ang II concentrations, contributing to further renal vasoconstriction and sodium reabsorption.^4^ Consequently, maternal plasma volume increases significantly from around 6 weeks’ gestation.

Normally, such extensive sodium retention would be expected to be accompanied by a reciprocal increase in the secretion of potassium, and a relative kaliuresis.^7^ However, pregnant women cumulatively retain some 300 to 350 mEq of potassium during normal pregnancy.^8^ There is a paucity of data on potassium homeostasis during normal pregnancy, let alone during pregnancies complicated by gestational hypertension (GH). One early study suggested that the kidneys of pregnant women become refractory to the kaliuretic effects of increased RAS and estrogen, perhaps due to an increased sensitivity to progesterone.^9^ To our knowledge, no clinical studies have reported associations between potassium homeostasis during pregnancy in women with GH alone and/or in women with proteinuric GH (preeclampsia).

Preeclampsia affects 2% to 8% of pregnant women worldwide and is one of the top 3 causes of maternal death.^10^ Perturbations of activity of the RAS in preeclampsia have been noted for >50 years,^11^ but research into the role of a dysfunctional RAS in preeclampsia has been limited. ACE inhibitors are well‐known teratogens in animal models^12^ and in humans^13^, ^14^ and are contraindicated in later pregnancy because of their serious adverse effects on the fetus.^15^ Nevertheless, Zhou et al recently showed that oxidation of circulating AGT is associated with increased generation of Ang I,^16^ and the proportion of circulating oxidized AGT is increased in pre‐eclamptic pregnancies.^16^

A number of other mechanisms exist by which not only circulating and/or local placental RAS but also an inappropriately activated intrarenal RAS could initiate or contribute to the pathology of preeclampsia through, for instance, angiogenic factors,^17^ the immune^18^ and coagulatory systems, and the complement system.^19^

To date, no correlation between plasma and urinary AGT in pregnant women has been shown, suggesting that autonomous regulation of the renal RAS occurs during pregnancy as it does in the nonpregnant state. Urinary AGT has been shown to reflect the rate of intrarenal synthesis and can be regarded as a biomarker of intrarenal RAS activity^20^; levels of the marker may also predict acute kidney injury and failure.^21^

In this study, for the first time, we examined urinary AGT, sodium, and potassium in normotensive and hypertensive pregnancies as a potential biomarker of intrarenal RAS activity, related to glomerular swelling as a sign of endotheliosis. In a limited number of kidney biopsies from this cohort,^22^ we confirmed the presence of intrarenal AGT by immunohistochemistry. Evidence of an important role for the inappropriate activation of intrarenal RAS in the pathophysiology of preeclampsia could indicate novel management and postnatal therapeutic strategies and targets to prevent short‐term and long‐term renal injury.

## Methods

The data and analytic methods, but not study materials, will be available to other researchers for purposes of reproducing the results or replicating the procedure. Material is not freely available because of the limited amounts and potential degradation.

### Sample Collection

Fully informed, signed consent for participation was obtained from all women, as previously described,^22^ following the ethics committee approval of the study by the University of Lund, and all procedures involving the participants were in accordance with the Helsinki Declaration of 1975. GH was defined as diastolic blood pressure >90 mm Hg, determined on >2 occasions >4 hours apart and arising after 20 weeks of gestation. Preeclampsia was defined as for GH but also including proteinuria (urine protein concentration >3000 mg/L in 2 random clean‐catch midstream specimens collected ≥4 hours apart). No woman had any known underlying renal or hypertensive disease before 20 weeks’ gestation.

Overnight urine samples were taken 1 to 3 days before renal biopsy in women with newly diagnosed GH admitted to Lund University Hospital from November 1999 to June 2001. All samples were collected before beginning antihypertensive treatment. Healthy pregnant women with comparable gestational age who were recruited from maternal healthcare centers in the catchment area of Lund University Hospital provided overnight urine samples as controls. Samples with evidence of urinary tract infection on urinalysis were excluded from all analyses. All samples were stored at −80°C in aliquots until analysis. Urine samples were available from 32 participants (10 normotensive controls, 6 women with GH, and 16 pre‐eclamptic women).

Renal biopsies were obtained as described previously.^23^ Briefly, biopsies were collected by an experienced radiologist according to a standard procedure. The sample was taken with a 1.2‐mm (outside diameter) needle using a Bard biopsy device under ultrasound guidance. Each biopsy, ≈20 mm in length, was immediately submerged in 0.9% sodium chloride solution and processed, with 1 part fixed in neutral formaldehyde 4% for immunohistochemical analysis.^23^

### Biochemical Assays

Urinary AGT concentrations were measured in duplicate by ELISA, following the manufacturer's protocol. Urine samples were diluted 1:10 using assay buffer. Intra‐ and interassay coefficients of variation were 4.4% and 5.6%, respectively. The mean urinary AGT concentration for each woman was normalized to urinary creatinine. Urinary volume and creatinine concentrations were measured in the Clinical Biochemistry Laboratory at Lund University by standard clinically validated methods. Urinary potassium and sodium concentrations were measured by inductively coupled plasma mass spectroscopy, as described previously, with specific standards.^24^, ^25^ All analyses were conducted as blinded to pregnancy outcome.

### Glomerular Volume

Mean glomerular volume had been estimated previously in biopsies containing at least 6 glomerular sections by a computer‐assisted stereological algorithm, as detailed previously.^22^

### Immunohistochemical Analysis From Matched Renal Biopsies

Only a limited amount of renal tissue remained from the biopsies after previous evaluations and analyses,^23^ and samples were used opportunistically for a preliminary evaluation of the immunohistochemical distribution of AGT within the nephron. Three biopsies contained glomeruli (2–4), but no biopsies contained sufficient vasculature for meaningful analysis. Protein expression in the kidney was analyzed by immunohistochemistry using the Dako Envision staining kits, as described previously,^26^ with rabbit anti‐AGT (0.6 mg/mL; HPA0031557 [Sigma Prestige]). For immunohistochemistry, a semiquantitative scoring system was applied, as described by Roy‐Chaudhury et al.^27^ In brief, the scale was as follows: 0=no specific staining, 0.5=very weakly positive, 1=weakly positive, 2=moderately strongly positive, 3=strongly positive. Scoring was generally influenced by the extent rather than the intensity of staining (ie, higher scores were given for widespread staining as opposed to evidence of localized intense staining).

### Statistical Analysis

Data were first assessed for distribution before analysis using SPSS (v24; IBM Corp). Urinary data were not normally distributed when all 3 groups (controls, GH, pre‐eclamptic pregnancies) were initially combined and thus are presented as median (interquartile range [IQR]; 25–75% quartiles] unless otherwise stated. Kruskal–Wallis nonparametric ANOVA was used throughout the study, with the null hypothesis being rejected at *P*<0.05. Linear correlations were computed using Spearman ρ. The association of urine potassium with AGT concentration was evaluated after log_10_ normalization. Curve characteristics were determined by regression analysis (GraphPad Prism, v6).

## Results

### Demographics of the Study Population

Table summarizes the basic demographic and pregnancy outcome data.

**Table 1:** Patient Demographics, BP, and Proteinuria and Urinary Sodium, Potassium, and Albumin Concentrations in NCs and Women With GH or Preeclampsia

Parameter	NC (n=10)	GH (n=6)	Preeclampsia (n=16)
Maternal age, y	29 (27–34)	28 (28–30)	30 (26–36)
BMI at booking, kg/m^2^	22.1 (21.3–22.3)* ^,^ †	25.9 (25.4–26.5)*	25.7 (24.3–30.3)†
After clinical diagnosis			
Highest systolic BP	130 (120–130)* ^,^ †	145 (140–150)* ^,^ ‡	160 (160–180)† ^,^ ‡
Highest diastolic BP	75 (75–85)* ^,^ †	110 (95–110)*	110 (107–118)†
Proteinuria, g/L	···	146 (84–251)‡	931 (701–1814)‡
PCR, mg/mmol	···	13.2 (5.8–22.5)‡	151.5 (77.3–270.9)‡
Gestation at delivery, d	281 (274–288)* ^,^ †	257 (254–265)‡	244 (233–256)†
Birth weight, g	3655 (3430–3890)* ^,^ †	2935 (2865–3205)*	2630 (2030–2840)†
Sodium, mg/mmol	33.3 (30.3–65.4)* ^,^ †	325.1 (194.1–429.5)*	344.2 (168.6–471.4)†
Potassium, mg/mmol	18.9 (15.2–32.8)* ^,^ †	216.8 (194–292.2)*	222.9 (165.9–288.5)†
Albumin, mg/mmol	0.1 (0.02–0.2)* ^,^ †	10.1 (7.5–13.4)* ^,^ ‡	59.7 (29.3–176.8)† ^,^ ‡

Data are presented as median (interquartile range) for continuous variables. Urinary parameters are normalized to creatinine levels. Groups were compared by Kruskall–Wallis. BMI indicates body mass index; BP, blood pressure; GH, gestational hypertension; NC, normotensive control; PCR indicates protein:creatinine ratio.

* *P*<0.05 between NC and GH.

† *P*<0.05 between NC and preeclampsia.

‡ *P*<0.05 between GH and preeclampsia.

### Urinary AGT Assay

**Figure 1:**
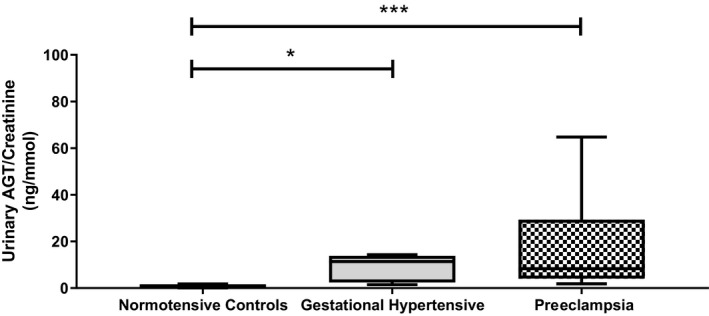
Urinary AGT (angiotensinogen) concentrations in normotensive control, gestational hypertensive, and pre‐eclamptic women. Data normalized to creatinine levels and presented as median (interquartile range). *P<0.05; ***P<0.0001.

Urinary AGT was significantly elevated in hypertensive pregnancies compared with normotensive controls (in nanograms of AGT per millimole of creatinine; normotensive control: 0.6 [IQR: 0.4–0.8]; GH: 11.3 [IQR: 2.8–13.6]; preeclampsia: 8.4 [IQR: 4.2–29.1]; Kruskal–Wallis, *P*<0.0001; Figure 1). Post hoc Mann–Whitney tests showed highly significant differences between normotensive controls and women with either GH (*P*=0.002) and preeclampsia (*P*<0.0001). The difference between women with GH and preeclampsia was not significant (*P*>0.3).

The patients’ blood pressures were measured at varying times throughout pregnancy. We considered only the highest recorded systolic and diastolic pressures for each patient in relation to urinary AGT output at the time and during the days of sampling. Regression analysis revealed highly significant associations between log_10_ urinary AGT/creatinine and maximum systolic (*r*=0.623; *P*<0.0001) and diastolic (*r*=0.787; *P*<0.0001) pressures. Furthermore, including log_10_ urinary potassium/creatinine output in the analysis with diastolic pressure significantly (*P*<0.02) improved *r* to 0.829, whereas adding in urinary sodium/creatinine output was without effect.

**Figure 2:**
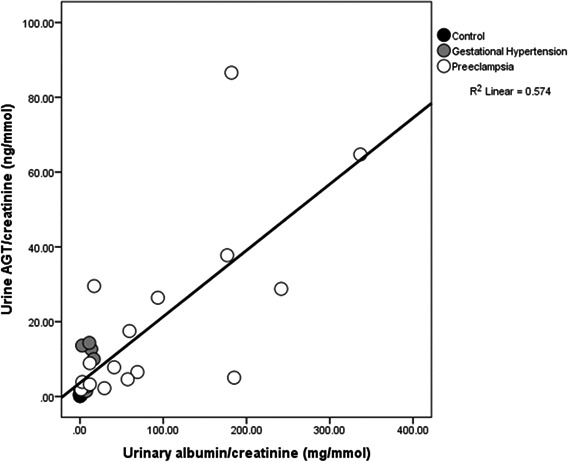
Scatterplot illustrating a strong positive relationship between urinary AGT (angiotensinogen) concentrations and albuminuria (*r*=0.76; *P*<0.0001).

Severity of preeclampsia is partly defined by the level of proteinuria/albuminuria.^28^, ^29^ Figure 2 shows a strong linear association between albuminuria and urinary AGT (*r*=0.76; *P*<0.0001) across the groups.

### Urine Composition

**Figure 3:**
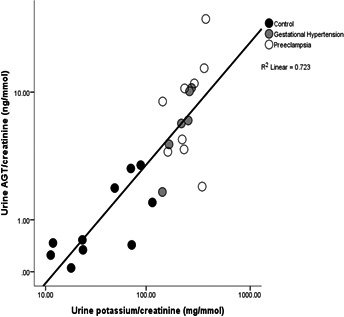
Scatterplot illustrating a clear positive relationship between urinary AGT (angiotensinogen) and potassium in all groups collectively (*r*=0.86; *P*<0.0001). Data normalized to creatinine levels and presented in log scale.

Urinary sodium and potassium concentrations were significantly higher in both hypertensive groups compared with the normotensive controls (*P*<0.001 for all; Table). Figure 3 shows a highly significant positive relationship between urinary potassium concentration and AGT in all groups (*r*=0.86; *P*<0.0001). No such association was identified between urinary sodium concentration and AGT (*P*>0.1).

### Glomerular Volume Calculations

**Figure 4:**
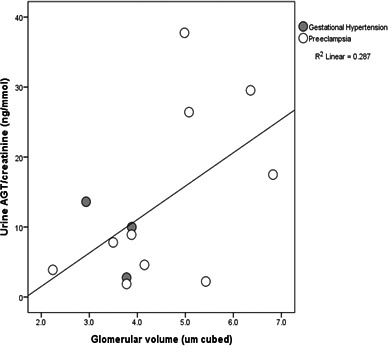
Scatterplot illustrating the positive relationship between urinary AGT (angiotensinogen) concentrations and estimated mean glomerular volume (μm^3^) in biopsies containing at least 6 glomerular sections, previously measured by a computer‐assisted stereological algorithm.^22^ Sample size was too small for statistical analysis.

Glomerular volume estimation was available only for women with GH (n=3) and preeclampsia (n=10). Figure 4 shows that the higher glomerular volumes were associated with higher urinary AGT, but the sample size was too small to allow statistical analysis.

### Immunohistochemical Analysis

**Figure 5:**
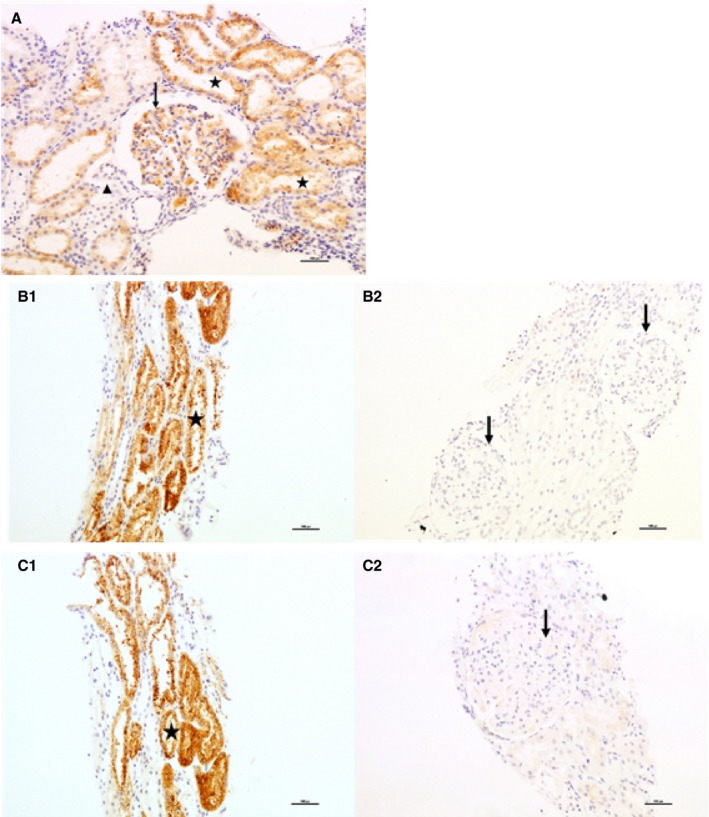
Immunohistochemical staining (×200 magnification) analysis of AGT (angiotensinogen) in kidney biopsies. **A**, Healthy control. Strong cytoplasmic staining (score: 3) is seen in >50% of proximal tubuli (asterisks in lumen) and, similarly, strong cytoplasmic staining (score: 3) in glomerular mesangium (arrow). A small distal tubule/collecting duct shows no staining (score: 0; above arrow head). **B**, Patient with gestational hypertension. (B1) Strong cytoplasmic staining (score: 3) is seen in >70% of proximal tubuli (asterisk in lumen). (B2) Two glomeruli are present showing no expression (arrows). **C**, Patient with severe preeclampsia. (C1) Strong cytoplasmic staining (score: 3) is seen in >70% of proximal tubuli (asterisk in lumen). (C2) One glomerulus is present showing very weak cytoplasmic staining (score: 0.50) in the mesangial area (arrow).

Inevitably, only limited tissue blocks were available from the previous study,^23^ and glomeruli were identifiable only in sections from 3 patients: fortuitously, one was a normotensive control, one had GH, and one had severe early onset preeclampsia. As Figure 5A shows, AGT protein was widely distributed throughout the glomerulus and in the proximal tubules but not in the distal tubules in the normotensive control. In contrast, Figure 5B2 and 5C2 show the absence of AGT protein in glomeruli from the GH and preeclampsia patients, although Figure 5B1 and 5C1 demonstrate AGT in the proximal tubules. Proximal and distal tubules were identifiable in sections from 9 normotensive controls, 6 patients with GH (Figure 5B1), and 16 pre‐eclamptic participants (Figure 5B2). Staining at level 2 or 3 was present in at least half of the sections in which proximal tubules were identified in all groups; no staining was observed in distal tubules from any patient.

## Discussion

We have shown, for the first time, that urinary AGT—a marker of activated intrarenal RAS—is significantly increased in women with both GH and preeclampsia. Although levels did not significantly differ by type of hypertension, we found a positive relationship with glomerular volume, indicating glomerular endotheliosis. There were also strong associations between urinary AGT and blood pressure across the study group as a whole. Ang II is a potent systemic vasoconstrictor; possible mechanisms linking intrarenal generation of Ang II and blood pressure have been reviewed previously.^30^ A strong association between urinary AGT and albumin excretion (Figure 2) was also demonstrated, although a causative relationship is impossible to determine from an observational study. However, it has been shown that the infusion of Ang II significantly increases albumin excretion in late‐pregnant spontaneously hypertensive rats.^31^ In nonpregnant humans, increased RAS activity is associated with increased proteinuria.^32^ Glomerular basement membrane “leakiness” might allow the passage of both albumin (molecular weight: 66.5 kDa) and AGT (molecular weight: 62 kDa). However, kidney podocytes, which form a filtration barrier against protein loss, are functionally disorganized in glomerular disease and in vitro by Ang II. Blockade of Ang II receptors in nonpregnant animal models of nephropathy results in improved function through reduced methylation of the nephrin promoter and decreased proteinuria.^33^ Human studies, both in vitro and observational, also suggest an intrarenal role for Ang II in proteinuria.^34^

Renin was first identified as an enzyme synthesized in the kidney, which was thought to be the only site of production until other autonomous and tissue‐specific RASs were discovered >50 years ago.^35^ AGT, the substrate for renin, is primarily synthesized in the liver, but mRNA for AGT has been identified at other sites, including the renal tubule.^36^ In the kidney, renin is released in response to physiological stimuli that normally activate the RAS, such as a decrease in tubular delivery of sodium chloride to the macula densa. Ordinarily, although the reaction of renin with AGT is rate limited by renin concentration, following first‐order kinetics, sufficient circulating concentrations of AGT (which are close to the Michelis–Menten constant for the reaction^37^) prevent any such limitation being reached. However, under circumstances that result in longer term RAS activation, such as treatment with estrogen‐based contraceptives or during pregnancy, AGT may become rate limiting.^38^ By 11 weeks’ gestation, an additional high‐molecular‐weight form of AGT is identifiable in the plasma of pregnant women.^39^ Moreover, the proportion of high‐molecular‐weight AGT to normal AGT is also increased in women who develop pregnancy‐associated hypertension,^40^ suggesting a means by which continued RAS activation might underpin GH.

AGT formed locally in the kidney in proximal tubular cells can spill over into the tubular lumen and be excreted in urine, where it can be measured as an indicator of internal RAS activity.^41^ ACE, which is responsible for the conversion of Ang I to Ang II, exists both bound to vascular endothelial cells and in a circulating form, allowing for both local and systemic generation of Ang II. ACE is also present in renal proximal tubules,^4^ confirming the plausibility of a renal RAS tailoring local generation of Ang II and capable of fine‐tuning intrarenal sodium reabsorption independent of the classical systemic RAS. This concept is supported by the observation that sodium depletion in Wistar Kyoto rats enhances renal AGT mRNA expression without a parallel change in renin expression.^42^ Intrarenally generated Ang II, by binding to AT1R, can cause direct renal vasoconstriction, altering tubuloglomerular feedback and increasing proximal tubular sodium reabsorption.^43^, ^44^ AT1R‐mediated uptake of circulating Ang II and augmentation of AGT expression in proximal tubular cells further augments intrarenal Ang II concentration and function, as does the AT1R‐mediated renin secretion from collecting ducts.^41^

Investigations of a role for the intrarenal RAS and, in particular, for AGT during pregnancy are in their infancy,^3^ although many roles both in pregnancy and preeclampsia have been suggested for the placental and circulating RAS.^45^

Ang II has a variety of roles in the body; not only is it one of the most potent circulating vasoconstrictors known, it also, paradoxically, stimulates synthesis and/or release of the main tonic vasodilator nitric oxide. Ang II promotes sodium resorption, both directly across the proximal tubule and indirectly by activating aldosterone, and stimulates vasculogenesis and angiogenesis, among other effects.^46^ The role of circulating RAS in preeclampsia remains controversial, however, and its suppression has been demonstrated in established hypertensive diseases of pregnancy (eg^47^). Sensitivity to exogenous Ang II is reduced in normotensive pregnancy but remains high in pre‐eclamptic women,^48^, ^49^ whereas sensitivity to other vasoconstrictors remains unchanged. This difference is apparent before the onset of preeclampsia.^50^

The finding of a strong association between urinary potassium and AGT excretion is shown in Figure 3. Ang II stimulates aldosterone synthesis and thus distal tubular sodium retention in exchange for potassium; therefore, potassium excretion rises. In normal pregnancy, the RAS is activated early; plasma aldosterone rises and some 950 mEq of sodium are retained,^8^ much of which is associated with the increase in plasma volume needed for the normal progress of pregnancy. However, the pregnant woman also retains some 450 mEq of potassium, although the mechanism for this has not yet been identified. In hypertensive pregnancy, plasma volume expansion is reduced.^51^ Global overexpression of AGT in transgenic mice was associated with a fall in plasma volume from midpregnancy.^52^ Although data from animal models must be viewed with caution, these data suggest the intriguing possibility that there may be prepregnancy or early pregnancy differences in the RAS that predispose a woman to developing preeclampsia. It follows that the increased renal synthesis of AGT that we report here in hypertensive pregnancy could be a response to relative hypovolemia in these women, although this was not measured in our study. Increasing dietary potassium intake in healthy volunteers has been associated with a fall in urinary AGT excretion.^53^ Due to limitations in sample size we were not able to compare fractional clearance of sodium and potassium in women with preeclampsia in this material, although it has been reported that renal hemodynamic measurements are on average 25% to 30% lower in women with preeclampsia than in gestation age–matched, healthy, normotensive pregnant women.^54^ The urinary renin concentration is known to be low.^20^, ^55^ Stored volumes of urine were too small to allow renin to be measured in this study.

In the limited amount of renal tissue that remained from biopsies after previous investigations,^23^ we opportunistically chose to demonstrate the presence of renal AGT expression by immunohistochemistry. We showed that in human pregnancy, AGT expression seems to be confined to the proximal tubule; no staining was demonstrated in any distal tubular segment. This result was also shown in biopsy material from nonpregnant patients,^56^ mice,^52^ and rats.^43^ The complete absence of glomerular AGT staining in hypertensive pregnancy, in contrast to normotensive control, is of interest and worthy of further study in appropriate animal models.

Measures of mean glomerular volume were available for 13 hypertensive women, having been estimated stereologically in renal tissue. Larger glomeruli were found in women with higher urinary AGT excretion, as shown in Figure 4. Glomerular volume is known to be increased in proportion to the severity of preeclampsia.^57^ The glomerular endotheliosis of preeclampsia has been ascribed to a decrease in circulating VEGF (vascular endothelial growth factor), itself a consequence of increased soluble Flt‐1 (fms‐like tyrosine kinase 1).^58^ Ang II stimulates soluble Flt in response to placental ischemia in rats.^17^ Ang II immunostaining in glomeruli has been shown in renal biopsies of patients with chronic kidney disease, in whom an association between urinary AGT and proteinuria also was noted.^59^ In these samples, we observed a strong positive correlation between urinary AGT and albuminuria (Figure 2), presumably due to podocyte and glomerular endothelial damage disrupting the filtration barrier of both.

There are a number of mechanisms by which an inappropriately activated intrarenal RAS could initiate or contribute to the pathology of preeclampsia, for instance through angiogenic and antiangiogenic factors,^45^ the immune system,^18^ and coagulation systems and the complement system.^19^ An activated complement system has been implicated in the pathophysiology of preeclampsia by an increasing body of evidence and can play a major role in causing renal injury also in other complement‐mediated renal diseases.^19^, ^60^ Renin can cleave C3 into C3a and C3b, thus activating the complement system by this alternative pathway.^60^ Targeting excessive complement activation has been suggested as a possible therapeutic strategy in prevention or treatment of preeclampsia^19^ and might also prevent long‐lasting damage to the kidney. Nevertheless, the risk of disrupting homeostatic functions and host defense cannot be disregarded.

Women with a history of preeclampsia have not only a 2‐fold increased risk of developing long‐term cardiovascular disease but also a 5‐ to 12‐fold increased risk of developing end‐stage renal disease,^61^ and there is need for renoprotective strategies to reduce late disease burden. In a paper highlighting the fact that World Kidney Day and International Women's Day coincided in 2018,^62^ the World Kidney Day Steering Committee emphasized that chronic kidney disease affects 10% of the world's adult population and takes place among the top 10 causes of death worldwide. With the knowledge that pregnancy is the most common cause of acute kidney injury in women of childbearing age,^60^ the urgency to monitor disease progression in preeclampsia, possibly by urinary AGT as a marker of inappropriately activated intrarenal RAS, is underscored. A timely delivery, also for renoprotective reasons, followed by possible therapeutic measures to prevent further renal injury could decrease women's risk for future renal and cardiovascular disease.

To the best of our knowledge, this study is the first to investigate, both functionally and histologically, intrarenal AGT in normal and hypertensive human pregnancies. While accepting the limitations on availability of renal biopsy specimens, similar samples are unlikely ever to be obtained again. The renal biopsy study in which these samples were procured showed no renal disease as an underlying cause of preeclampsia. They also showed the typical lesion of “endotheliosis” to be present in some normal pregnant controls, thus no clinical indication remains for performing a renal biopsy to diagnose renal disease or preeclampsia in pregnancy in either a clinical or research setting.

These results indicate the presence of intrarenal AGT in renal tissue from women with GH and preeclampsia and increased urinary AGT and potassium excretion in our unique set of matched urine samples. The findings suggest inappropriately increased intrarenal RAS activation, seemingly associated with glomerular swelling as an indication of endotheliosis. These changes are likely to be independent of the systemic renin‐angiotensin status. An inappropriately activated RAS can lead to renal injury through renal vasoconstriction and numerous other mechanisms, including activation of the complement system. Apart from a timely delivery, other therapeutic measures should be considered to limit damage to the maternal kidneys. Even if treatment were available only in the immediate postpartum period, it might attenuate the risk of future renal disease.

## Sources of Funding

This work was produced by Mistry under the terms of a British Heart Foundation Basic Science Intermediate Basic Science Fellowship (FS/15/32/31604) and an European Renal Association‐European Dialysis and Transplant Association Long‐Term Fellowship (LTF 137‐2013). Mistry, Kurlak, and Broughton Pipkin received an International Collaboration Grant from the University of Nottingham to travel to Lund during this study. Strevens received a travel research grant from the Faculty of Medicine, Lund University (Carl Swartz Minnesfond).

## Disclosures

None.
